# Association between urinary phthalate levels and chronic pain in US adults, 1999–2004: A nationally representative survey

**DOI:** 10.3389/fneur.2023.940378

**Published:** 2023-02-02

**Authors:** Guoping Jin, Yaoyao Nie, Jiayao Fan, Ye Yang, Dingwan Chen, Yingjun Li, Li Ju

**Affiliations:** ^1^Department of Orthopaedics, Ningbo No. 9 Hospital, Ningbo, Zhejiang, China; ^2^School of Public Health, Hangzhou Medical College, Hangzhou, China; ^3^School of Public Health and The Second Affiliated Hospital, Zhejiang University School of Medicine, Hangzhou, China; ^4^Department of Occupational and Environmental Health, School of Public Health, Hangzhou Medical College, Hangzhou, China

**Keywords:** phthalates, chronic pain, NHANES, epidemiology, plasticizers

## Abstract

**Introduction:**

Chronic pain is a public health concern throughout the world. Ascertaining and managing its risk factors helps develop well-directed treatment plans and prevention strategies. Phthalates (PAEs) exposure leads to various health problems. The present study aims to explore the potential correlation between urinary PAEs metabolites and chronic pain in adults.

**Methods:**

The study population data were extracted from the National Health and Nutrition Examination Survey (NHANES) conducted from 1999 to 2004 in the United States. Seven urinary PAEs metabolites were used to assess long-term PAEs exposure. The assessment of chronic pain was determined by a self-report questionnaire. Weighted analyses were conducted to consider the complex sampling design. Models were adjusted by demographic data and lifestyle factors. Urinary PAEs metabolites were assessed as both continuous and categorical variables. Tertile 1 was considered as the reference. Stratified analyses were performed by gender and pain site. All data analyses were conducted with STATA, version 15.1. *P* < 0.05 was considered with statistical significance.

**Results:**

A total of 4,196 participants were considered in our final analysis. Chronic pain prevalence reached 52.19% (*n* = 2,138) among the participants, with women accounting for a large proportion (57.75% vs. 42.25%). After multivariable logistic regression analysis, a higher prevalence of chronic pain was observed among participants in the third tertile of mono-(2-ethyl)-hexyl phthalate (MEHP) (OR = 1.23, 95% CI = 1.02–1.48, *P* = 0.034) and mono-benzyl phthalate (MBzP) (OR = 1.28, 95% CI = 1.04–1.58, *P* = 0.022) in our adjusted model. The logtransformed concentration of MBzP also showed a significant association with chronic pain prevalence (OR = 1.09, 95% CI = 1.01–1.18, *P* = 0.036) in the adjusted model. In further analysis, the positive correlations of urinary phthalate metabolites with chronic pain remained robust when stratified by gender and chronic pain site.

**Conclusions:**

Our findings presented a positive correlation between urinary PAEs metabolites and chronic pain among adult participants, and more causal research should be conducted to ascertain the interactions between the two and to expound their underlying mechanisms.

## 1. Introduction

Chronic pain is defined as pain that prolongs for over 3 months. It causes major suffering and has a weighted mean prevalence of 20% in adults, being more common in women, older people, and populations with health deprivation (1). Chronic pain leads to many physical and emotional burdens beyond the pain itself, including disability, emotional disturbance, sleep deprivation, and poor quality of life, etc. (1, 2). The financial cost is enormous to society and is estimated to be over 200 billion euros each year in Europe and 150 billion dollars each year in the US (3). The World Health Organization (WHO) recognizes chronic pain as a worldwide public health concern.

Factors contributing to chronic pain include genetics, older age, lower cultural education, and lower socioeconomic status (4, 5). Other modifiable factors include smoking status, alcohol intake, nutrition, and occupational characteristics, etc. (4, 5). It is necessary to know the risk factors of how chronic pain initiates and progresses for the further development preventive and management strategies.

Phthalates (PAEs), esters of 1,2-benzene dicarboxylic acid, are synthetic organic chemicals commonly used in industrial or consumer applications as plasticizers. PAEs are easily chipped off products and become contaminants in the workplace, indoor air, or food (6–8). Subsequently, people are daily exposed to them by inhalation, oral intake, or skin contact. In a cross-sectional survey, most people had detectable PAEs metabolites in urine, including di-(2-ethylhexyl)-phthalate (DEHP), diethyl phthalate (DEP), and di-n-butyl phthalate (DBP) (9). With this constant exposure, various human adverse health outcomes occur. Studies showed PAEs exposure led to reproductive toxicity, developmental toxicity, and cardiovascular disorders (10–12). Exposure to PAEs could also affect the pathogenesis of endometriosis (13). Our previous studies showed an association between PAEs with sarcopenia and osteoarthritis (14, 15). However, there is much less information about the association between PAEs and chronic pain. Usually, chronic pain is a common concomitant symptom of another severe disease, such as coronary heart disease, endometriosis, and osteoarthritis, as mentioned above. More than that, chronic pain has been recognized as an independent condition with its own taxonomy and definitions (16). Thus, the question of whether there is direct pain after PAEs exposure.

To explore if PAEs are environmental risk factors of chronic pain in humans, a cross-sectional analysis was conducted in our present study using data from the National Health and Nutrition Examination Survey (NHANES) cohort conducted in 1999–2004 in the US. Several urinary PAEs metabolites were examined, and their correlation with chronic pain prevalence was deeply explored.

## 2. Methods

### 2.1. Study population

Population data were extracted from the NHANES, an authority organization to evaluate the nutritional and health status of the US population. General information, such as participants' demographic, socioeconomic, and physical activity and dietary habits were collected through in-home interviews by the Centers for Disease Control and Prevention (CDC). The quality assurance and quality control procedures ensured the validity and reliability of the questionnaire and were described in the field procedures manuals that can be found here https://wwwn.cdc.gov/nchs/data/nhanes/2005-2006/manuals/mec_interview.pdf. Biospecimens were collected in a specially equipped mobile examination center (MEC). The whole program was approved by the Ethics Review Committee of the National Center for Health Statistics. All participants signed a written consent after recruitment. For our study, we analyzed participants aged ≥20 years old for the cycles 1999–2000, 2001–2002, and 2003–2004 from the NHANES, which has available data both for PAEs metabolites as exposure and chronic pain as an outcome. Exclusion criteria for participants included no testing data on urinary creatinine concentrations (used to account for the variation of urine dilutions) and missing relevant covariates (analyzed in multivariable logistic regression models). Ethics for collecting original data were approved, and informed consent was obtained from all participants in the NHANES survey.

### 2.2. Measurement of urinary PAEs metabolites

Combined urine specimens were collected and kept frozen (-20°C) by the National Center for Environmental Health (NCEH). Seven urinary PAEs metabolites were tested, namely, mono-n-butyl phthalate (MnBP), mono-ethyl phthalate (MEP), mono-benzyl phthalate (MBzP), mono-(2-ethyl)-hexyl phthalate (MEHP), mono-cyclohexyl phthalate (MCP), mono-n-octyl phthalate (MOP), and mono-isononyl phthalate (MNP). Solid-phase extraction-high performance liquid chromatography-isotope dilution-tandem mass spectrometry (HPLC-MS/MS) was used in measuring PAEs metabolite levels. The limit of detection (LOD) of PAEs metabolites in 1 ml urine ranges from 0.072 to 1.68 ng/ml. A value of LOD divided by the square root of two was used when the levels were below LOD. All urinary PAEs metabolite levels were corrected by creatinine (μg/g creatinine) and measured by the Beckman Synchron CX3 clinical analyzer and the Roche/Hitachi Modular P chemistry analyzer (17, 18).

### 2.3. Assessment of chronic pain

The assessment of chronic pain was conducted by a self-report questionnaire. Neck pain, low back pain, headaches, or migraines during the past 3 months, and many other types of pain were recorded. In this context, pain refers to a pain lasting more than a whole day, and did not include minor aches. The participants were asked: “During the past 3 months, did you have neck pain?”. According to the answers, the participants who answered positively were divided into a chronic pain group, and those who did not have any kind of chronic pain were classified into the no chronic pain group.

### 2.4. Statistical analysis

Weighted analyses were conducted to consider the complex sampling design. Firstly, a descriptive analysis was incorporated to estimate between-group differences in baseline characteristics, using χ^2^ tests for categorical variables, whereas linear regressions were used for continuous variables. Secondly, multivariable logistic regression models with odds ratios (ORs) and corresponding 95% confidence intervals (CIs) were used to assess the associations between urinary PAEs metabolites and chronic pain. Models were adjusted by demographic data and lifestyle factors, including age, gender, ethnicity, education level, marital status, smoking status, body mass index (BMI), physical activity habit, diabetes mellitus, rheumatoid arthritis, osteoarthritis, and survey cycle. Ethnicity was divided into non-Hispanic white, non-Hispanic black, Mexican American, and others. Education level was categorized into three groups: under high school, high school or equivalent, and above high school. Marital status was categorized into married/cohabiting, widowed/divorced/separated, and never married. Smoking status was divided into never, currently, and ever. Physical activity in recreational time was divided into sedentary, insufficient, moderate, and high. BMI was categorized into normal (<25 kg/m^2^), overweight (25–30 kg/m^2^), and obesity (≥30 kg/m^2^). History of diseases (yes, no) was defined on the information from self-reported medical conditions.

Urinary PAEs metabolites were assessed as both continuous and categorical variables. Tertile 1 was considered as the reference. Stratified analyses were performed by gender and pain site. All data analyses were conducted with STATA, version 15.1. *P* < 0.05 was considered with statistical significance.

## 3. Results

### 3.1. Characteristics of participants

**Figure 1:**
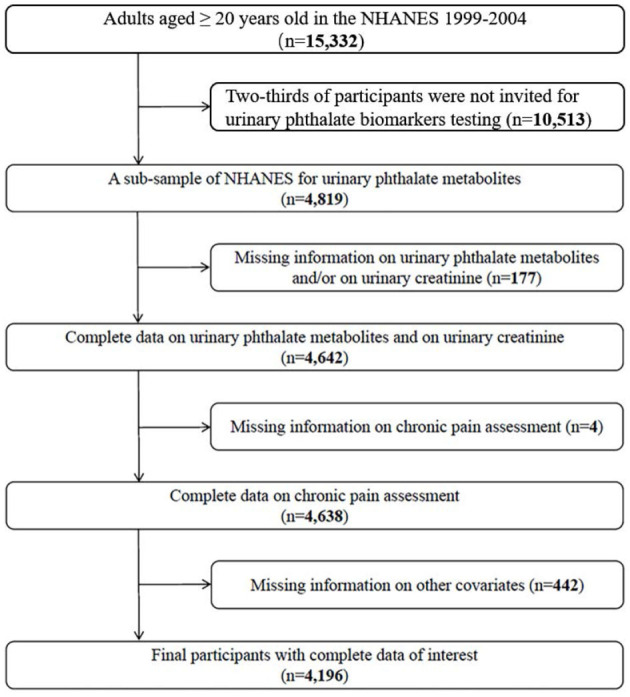
Flow chart of participants selection.

There were 15,332 adults aged over 20 years old in the NHANES 1999–2004. Out of those, 4,819 were recruited to measure their PAEs metabolites in urine. Moreover, we further excluded some participants whose information on urinary PAEs metabolites, urinary creatinine (*n* = 177), chronic pain assessment (*n* = 4), and/or other related variates (*n* = 442) was not adequate. Finally, 4,196 participants were considered for our further analysis (Figure 1).

Table 1 shows all the demographic characteristics of the participants in the two groups. Among all of the 4,196 participants (1,968 men and 2,228 women), the average age was 44.56 years old for the chronic pain group and 46.07 years old for the control group (*P* < 0.001). Most of the participants were non-Hispanic whites (71.76%), married or cohabiting (64.37%), and had no history of diabetes mellitus (92.05%), rheumatoid arthritis (95.47%), or osteoarthritis (92.76%). Significant differences were found in education, smoking status, physical activity in recreational time, and history of rheumatoid arthritis or osteoarthritis between the control group and the chronic pain group. The weighted percentage of chronic pain reached 52.19% (*n* = 2,138), and women accounted for a large part of chronic pain sufferers (57.75 vs. 42.25%).

**Table 1:** Basic characteristics of participants in US adults, NHANES 1999–2004.

**Characteristics**	**Total** **(*n =* 4,196)**	**Control** **(*n =* 2,058)**	**Chronic pain** **(*n =* 2,138)**	**P-value^a^**
Age (years, mean±SD)	45.28 ± 21.20	46.07 ± 02.98	44.56 ± 51.02	<0.001
**Gender**, ***n*** **(%)**				
Male	1,968 (47.13)	1,082 (52.45)	886 (42.25)	<0.001
Female	2,228 (52.87)	976 (47.55)	1,252 (57.75)	
**Race/ethnicity**, ***n*** **(%)**				
Non-Hispanic white	2,125 (71.76)	1,022 (70.98)	1,103 (72.47)	0.292
Non-Hispanic black	780 (10.50)	374 (10.47)	406 (10.53)	
Mexican American	952 (7.19)	489 (7.78)	463 (6.66)	
Others	339 (10.55)	173 (10.77)	166 (10.34)	
**Education**, ***n*** **(%)**				
Under high school	1,343 (20.22)	621 (17.76)	722 (22.47)	0.001
High school or equivalent	950 (25.17)	444 (24.06)	506 (26.18)	
Above high school	1,903 (54.61)	993 (58.18)	910 (51.35)	
**Marital status**, ***n*** **(%)**				
Married/cohabiting	2,631 (64.37)	1,306 (65.14)	1,325 (63.67)	0.052
Widowed/divorced/separated	917 (18.49)	419 (16.45)	498 (20.36)	
Never married	648 (17.14)	333 (18.41)	315 (15.97)	
**BMI**, ***n*** **(%)**				
Normal	1,275 (32.56)	654 (33.89)	621 (31.34)	0.056
Underweight	58 (1.75)	25 (1.45)	33 (2.03)	
Overweight	1,482 (32.94)	738 (33.24)	744 (32.66)	
Obese	1,381 (32.75)	641 (31.42)	740 (33.97)	
**Smoking status**, ***n*** **(%)**				
Never	2,191 (51.03)	1,120 (54.10)	1,071 (48.21)	<0.001
Current	920 (24.90)	382 (20.02)	538 (29.38)	
Ever	1,085 (24.07)	556 (25.88)	529 (22.41)	
**Physical activity in recreational time**, ***n*** **(%)**				
Sedentary	1,784 (34.35)	834 (31.90)	950 (36.60)	0.003
Insufficient	869 (22.54)	410 (21.60)	459 (23.40)	
Moderate	513 (14.28)	264 (14.98)	249 (13.64)	
High	1,030 (28.83)	550 (31.51)	480 (26.36)	
**Diabetes mellitus**, ***n*** **(%)**				
Yes	459 (7.95)	220 (7.79)	239 (8.09)	0.612
No	3,737 (92.05)	1,838 (92.21)	1,899 (91.91)	
**Rheumatoid arthritis**				
Yes	235 (4.53)	76 (2.99)	159 (5.94)	<0.001
No	3,961 (95.47)	1,982 (97.01)	1,979 (94.06)	
**Osteoarthritis**				
Yes	310 (7.24)	107 (4.93)	203 (9.35)	<0.001
No	3,886 (92.76)	1,951 (95.07)	1,935 (90.65)	
**Survey**				
1999–2000	1,243 (29.92)	630 (30.26)	613 (29.60)	0.383
2001–2002	1,505 (34.39)	730 (34.85)	775 (33.96)	
2003–2004	1,448 (35.70)	698 (34.89)	750 (36.43)	

NHANES, National Health and Nutrition Examination Survey; BMI, body mass index; SD, standard deviation; n, numbers of subjects; %, weighted percentage. ^a^Difference in characteristics between control group and chronic pain group.

### 3.2. Correlations between urinary phthalate metabolites and chronic pain

Urinary PAEs metabolites were examined and are shown in Table 2, and LOD, geometric mean (GM) concentration with 95% CI, and tertiles of concentrations for seven urinary PAEs metabolites were calculated. Levels of urinary PAEs metabolites were all creatinine-corrected (μg/g creatinine). Consequently, MnBP, MBzP, and MEP were all detected in over 99% of the participants. The detection frequency was 65.26% for MEHP and <10% for MCP, MNP, and MOP. The concentration of MEP was the highest among all metabolites. We did further analysis using PAEs metabolites with a detection frequency of more than 60%, including MEP, MBzP, MnBP, and MEHP, with chronic pain among US adults.

**Table 2:** Distribution of urinary phthalates levels (μg/g creatinine) in US adults,NHANES 1999–2004.

	**≥LOD (%)^a^**	**GM (95% CI)^b^**	**Tertile 1^b^**	**Tertile 2^b^**	**Tertile 3^b^**
MnBP	99.24	186.3 (181.3, 191.4)	≤ 128.6	128.6–255.3	>255.3
MCP	8.43	5.3 (5.2, 5.5)	≤ 3.1	3.1–7.7	>7.7
MEP	100.00	1,326.7 (1,271.9, 1,384.0)	≤ 691.2	691.2–2,241.2	>2,241.2
MEHP	65.26	29.6 (28.6, 30.7)	≤ 17.2	17.2–44.8	>44.8
MNP	3.59	9.6 (9.4, 9.9)	≤ 6.3	6.3–11.7	>11.7
MOP	0.97	11.6 (11.4, 11.9)	≤ 7.7	7.7–14.2	>14.2
MBzP	99.45	80.4 (78.0, 82.9)	≤ 56.4	56.4–116.2	>116.2

NHANES, National Health and Nutrition Examination Survey; LOD, limit of detection; GM, geometric mean; CI, confidence interval. ^a^Unweighted proportions. ^b^Urinary levels of phthalate metabolites were all creatinine-corrected (μg/g creatinine), and the data were multiplied by 1000 to show more information.

After multivariable logistic regression analysis, a higher prevalence of chronic pain was observed among participants in the maximal tertile of MEHP (OR = 1.23, 95% CI = 1.02–1.48, *P* = 0.034) and MBzP (OR = 1.28, 95% CI = 1.04–1.58, *P* = 0.022) in the adjusted model. The log-transformed concentration of MBzP also showed a significant association with chronic pain prevalence (OR = 1.09, 95% CI = 1.01–1.18, *P* = 0.036) in the adjusted model (Table 3).

**Table 3:** Association of urinary phthalate metabolites and chronic pain among US adults, NHANES 1999–2004 (*n* = 4,196).

	**Model 1** ^**a**^		**Model 2** ^**b**^	
	**OR (95% CI)**	**P-value**	**OR (95% CI)**	**P-value**
**MnBP**				
Tertile 1	Reference	–	Reference	–
Tertile 2	**1.26 (1.04, 1.53)**	**0.022**	1.16 (0.97, 1.38)	0.112
Tertile 3	**1.35 (1.04, 1.75)**	**0.023**	1.13 (0.86, 1.50)	0.368
Log-transformed MnBP	**1.13 (1.01, 1.26)**	**0.029**	1.04 (0.93, 1.17)	0.463
**MEP**				
Tertile 1	Reference	–	Reference	–
Tertile 2	0.97 (0.81, 1.17)	0.758	0.92 (0.74, 1.14)	0.445
Tertile 3	0.98 (0.81, 1.19)	0.863	0.92 (0.74, 1.13)	0.419
Log-transformed MEP	1.02 (0.97, 1.08)	0.391	1.00 (0.94, 1.06)	0.969
**MEHP**				
Tertile 1	Reference	–	Reference	–
Tertile 2	1.10 (0.91, 1.32)	0.331	1.08 (0.89, 1.31)	0.434
Tertile 3	**1.25 (1.03, 1.50)**	**0.022**	**1.23 (1.02, 1.48)**	**0.034**
Log-transformed MEHP	1.07 (0.99, 1.15)	0.071	1.06 (0.98, 1.14)	0.124
**MBzP**				
Tertile 1	Reference	–	Reference	–
Tertile 2	**1.24 (1.04, 1.49)**	**0.018**	1.14 (0.96, 1.34)	0.137
Tertile 3	**1.54 (1.26, 1.88)**	**<0.001**	**1.28 (1.04, 1.58)**	**0.022**
Log-transformed MBzP	**1.17 (1.09, 1.27)**	**<0.001**	**1.09 (1.01, 1.18)**	**0.036**

NHANES, National Health and Nutrition Examination Survey; n, numbers of subjects; OR, odds ratio; CI, confidence interval. ^a^Logistic regression without adjustment. ^b^Logistic regression adjusted for age, gender, race/ethnicity, education level, marital status, BMI, smoking status, physical activity in recreational time, diabetes mellitus, rheumatoid arthritis, osteoarthritis, and survey cycle. Bold fonts highlight values with statistical differences.

### 3.3. Subgroup analysis

**Figure 2:**
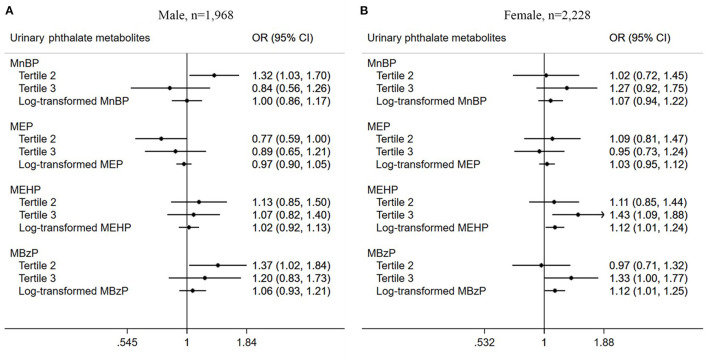
Association between urinary phthalate metabolites and chronic pain among US adults stratified by gender. **(A)** Male, *n* = 1,968; **(B)** Female, *n* = 2,228.

Subsequently, we did further subgroup analyses to discover more about chronic pain among US adults. Correlation of urinary PAEs metabolites with chronic pain stratified by gender was conducted, as shown in Figure 2. Male participants in the second tertile of MnBP (OR = 1.32, 95% CI = 1.03–1.70, *P* = 0.032) and MBzP (OR = 1.37, 95% CI = 1.02–1.84, *P* = 0.036) had higher chronic pain prevalence in the adjusted model. In contrast, participants in the second tertile of MEP showed a decreased prevalence of chronic pain (OR = 0.77, 95% CI = 0.59–1.00, *P* = 0.046). In female adults, those in the third tertile of MEHP showed an increased prevalence of chronic pain (OR = 1.43, 95% CI = 1.09–1.88, *P* = 0.012). The positive association between log-transformed MEHP (OR = 1.12, 95% CI = 1.01–1.24, *P* = 0.028) and MBzP (OR = 1.12, 95% CI =1.01–1.25, *P* = 0.041) with chronic pain prevalence was also found.

**Figure 3:**
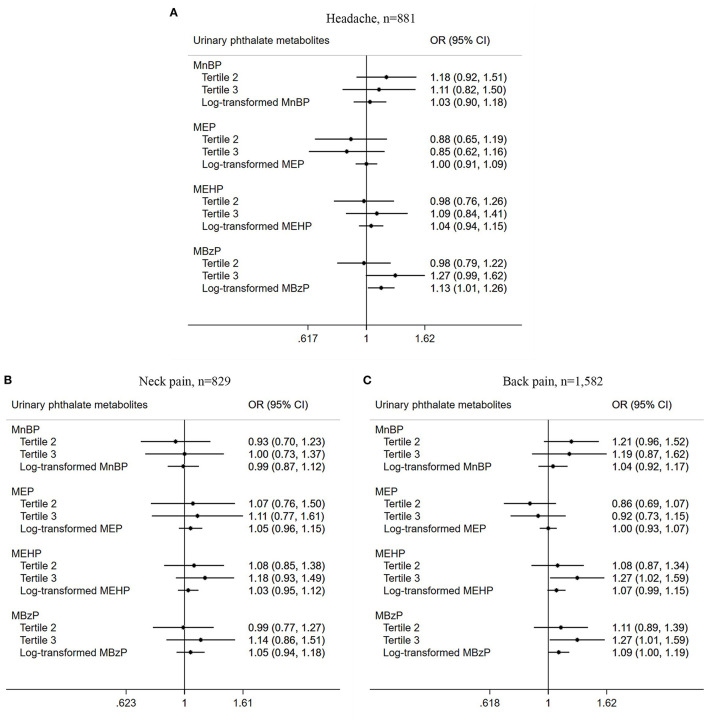
Association between urinary phthalate metabolites and chronic pain among US adults stratified by site. **(A)** Headache, *n* = 881; **(B)** Neck pain, *n* = 829; **(C)** Back pain, *n* = 1,582.

Association of urinary PAEs metabolites with chronic pain among US adults stratified by pain site was also conducted, and chronic pain in the head (*n* = 881), neck (*n* = 829), and back (*n* = 1,582) were included. As listed in Figure 3, there was a positive relationship between the third tertile of MEHP (OR = 1.27, 95% CI = 1.02–1.59, *P* = 0.041) and MBzP (OR = 1.27, 95% CI = 1.01–1.59, *P* = 0.042) with chronic back pain prevalence in the logistic regression adjusted model. Those with a higher log-transformed concentration of MBzP were found to be associated with chronic headache prevalence in the adjusted model (OR = 1.13, 95% CI = 1.01–1.26, *P* = 0.033).

## 4. Discussion

Chronic pain has been the cause of major suffering among adults and has become a global public concern. It is reported that 1.9 billion people suffer recurrent tension-type headaches (19). Lower back pain is a main cause of disability worldwide, featuring in the list of top five causes of disability (19). There is a need for more studies on chronic pain so that well-directed treatment plans and prevention strategies are developed. Ascertaining the chronic pain risk factors on its prediction, development, assessment, management, and prognosis, will help to deal with these predisposing factors and their impact on pain.

### 4.1. Risk factors of chronic pain

Currently, risk factors of chronic pain are demographic factors, clinical diseases, or lifestyle and behavior, such as smoking, vitamin D intake, etc. (20–24). Advanced age has a complex association with chronic pain. Older patients have higher morbidity of chronic pain than younger ones (23). Poor job control, lack of work autonomy, or job satisfaction are all occupational risk factors for chronic pain (25). In the present study, a novel recognition was provided regarding a positive correlation of PAEs exposure, especially for MBzP and MEHP, with chronic pain in US adults, according to the dataset from the NHANES 1999–2004. As shown in the results, individuals with a higher concentration of urinary MBzP, MEHP, and MnBP developed a higher prevalence of chronic pain after a multivariable adjustment analysis. The association remained robust when stratified by gender and pain site, both for male and female participants.

### 4.2. Biomonitoring of PAEs metabolites

PAEs are commonly used in personal care products, textiles, plasticizers, paints, and waxes at a rate of millions of tons each year, resulting in broad exposure for the general population. Likewise, studies show that rather than sociodemographic characteristics, some lifestyle determinants, such as the use of cosmetic products, are important predictors in the levels heavy metals in the urine of pregnant women (26–28). As PAEs are metabolized and excreted rapidly, the measurement of urinary PAEs metabolites, such as MBzP and MEHP, is a common approach to assess long-term PAEs exposure in epidemiology studies. In Darvishmotevalli's study, MBzP, MBP, MEHP, and MEHHP were detected in 100% of 121 Iranian pregnant women. Significant correlations were observed between these urinary PAEs metabolites with the use of plastic packaging, lower physical activity, and passive smoking exposure during pregnancy (29). Significant correlations were also observed between the urinary MBzP and MEHHP levels with the birth weight of female neonates, with MBP and MBzP having negative associations with the head circumference in male and female newborns (30). In our study, we analyzed the association between chronic pain and urine PAEs metabolites with their creatinine-corrected values to correct for the potential dilutions in combined urinary samples.

Urinary PAEs metabolite concentrations are different among countries. Data from Finland, Austria, Germany, Korea, and the United States were reported. The highest median level of urinary MEP was observed in Finland and the highest P95 value was registered in the US and Austria. Data from 2015 showed the lowest level of MEP in Germany. No MEP data was available from Korea (31). In our data collection, MEP concentration was the highest among all seven metabolites, and almost ten-fold of others. The median urinary MBzP concentration in Finland was the highest among these five countries, up to 10 times higher than that in Germany. At the same time, the median urinary MEHP concentration in Korea was the highest (31). In our study, concentrations of MBzP and MEHP were both at a high level.

For possible reasons of the variation, Europe has authorized the use of some PAEs since 2015 because of the reproductive toxicity. These regulatory actions partly affect the PAEs exposure of the European population (32). Another factor that also contributes to the variable concentrations is the fact that, in some countries newer PAEs or PAE substitutes are developed and replaced some older PAEs, including BBP, DnBP, and DEHP.

### 4.3. Mechanisms of PAEs exposure on chronic pain

**Figure 4:**
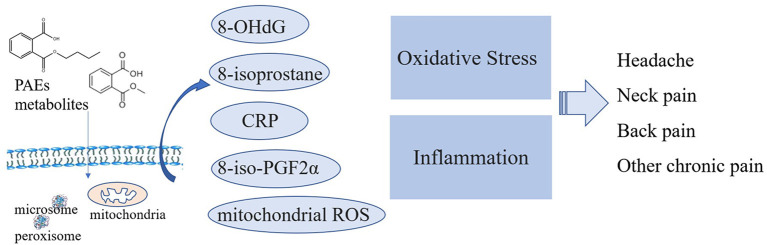
Plausible molecular mechanisms of PAE metabolites.

Mechanisms to explain the correlation of PAEs exposure with chronic pain are elusive. Some studies suggest a potential role of mitochondrial ROS and oxidative stress in neuropathic and inflammatory pain (33, 34). A slight increase of unspecific inflammatory markers, such as C-reactive protein (CRP), was associated with low back pain, hip/leg pain, and the number of pain sites, as shown in a follow-up study done over seven years (35). In comparison, PAEs exposure was also reported to increase oxidative stress markers (17, 36, 37). PAEs metabolites promote 8-iso-prostaglandin F2α (8-iso-PGF2α) expression both from oxidative stress and inflammation-derived sources (38). The relationships of 8-hydroxydeoxyguanosine (8-OHdG) were significant for MnBP, MBP, and mono-iso-butyl phthalate (MiBP), and the associations of 8-isoprostane with PAEs metabolites were also statistically significant for MBP and MiBP (17). Additionally, the urinary MBzP, MCPP, and MCNP levels were all related to an elevation in CRP (17, 39). MEHP induces a pro-inflammatory state in differentiated adipocytes (37). Accordingly, we proposed that PAEs exposure probably induced chronic pain *via* induction of oxidative stress or inflammation (Figure 4). However, the biological explanation of the association between PAEs exposure and chronic pain was still a hypothesis, and more experimental studies are needed.

## 5. Conclusion

The association between urinary PAEs metabolites and total chronic pain, as well as site-specific chronic pain, in US adults was first observed in the study, but more causal studies are needed to confirm our findings and to clarify the underlying mechanisms between PAEs exposure with chronic pain prevalence.

## Data availability statement

Publicly available datasets were analyzed in this study. This data can be found here: Centers for Disease Control and Prevention (CDC) National Health and Nutrition Examination Survey (NHANES), https://wwwn.cdc.gov/nchs/nhanes/Default.aspx.

## Ethics statement

The studies involving human participants were reviewed and approved by the Ethics Review Committee of the National Center for Health Statistics. Written informed consent to participate in this study was provided by the patient/participants or patient/participants' legal guardian/next of kin.

## Author contributions

LJ: conceptualization, funding acquisition, and writing—review and editing. YN: data curation and investigation. JF: formal analysis and methodology. YY: project administration and software. DC: supervision and validation. GJ: visualization and writing—original draft. YL: validation, data curation, and writing—review and editing. All authors have read and approved the final version.
